# Differentiation of multinodular and vacuolating neuronal tumor and dysembryoplastic neuroepithelial tumor based on MRI

**DOI:** 10.55730/1300-0144.5988

**Published:** 2024-12-30

**Authors:** Burcu GÜL, Bora KORKMAZER, Ahmet Kürşat KARAMAN, Esra KOÇHAN KIZILKILIÇ, Mahmut Esat AYKAN, Çiğdem ÖZKARA, Nil ÇOMUNOĞLU, Cihan İŞLER, Serdar ARSLAN, Osman KIZILKILIÇ

**Affiliations:** 1 Department of Radiology, Intermed Clinic, İstanbul, Turkiye; 2 Department of Radiology, Division of Neuroradiology, İstanbul University-Cerrahpaşa, İstanbul, Turkiye; 3 Department of Radiology, Süreyyapaşa Chest Diseases and Thoracic Surgery Training Hospital, İstanbul, Turkiye; 4 Department of Neurology, Cerrahpaşa Faculty of Medicine, İstanbul University-Cerrahpaşa, İstanbul, Turkiye; 5 Department of Radiology, Cerrahpaşa Faculty of Medicine, İstanbul University-Cerrahpaşa, İstanbul, Turkiye; 6 Department of Pathology, Cerrahpaşa Faculty of Medicine, İstanbul University-Cerrahpaşa, İstanbul, Turkiye; 7 Department of Neurosurgery, Cerrahpaşa Faculty of Medicine, İstanbul University-Cerrahpaşa, İstanbul, Turkiye

**Keywords:** Multinodular and vacuolating neuronal tumor, dysembryoplastic neuroepithelial tumor, magnetic resonance imaging, glioneuronal tumor, seizure

## Abstract

**Background/aim:**

To compare the MRI findings and clinical features of multinodular and vacuolating neuronal tumor (MVNT) and dysembryoplastic neuroepithelial tumor (DNET), and reveal the distinguishing features of these tumors from each other.

**Materials and methods:**

Patients with a suspected magnetic resonance imaging (MRI)-based diagnosis of MVNT between 2018 and 2022 were collected from the hospital database. In addition, patients diagnosed with DNET on histopathological examination and who had MRIs in the same time period were included in the study. The MRI findings and clinical features were evaluated for each patient.

**Results:**

There were 21 patients in the MVNT group and 20 patients in the DNET group. Headache was the most common symptom in patients with MVNTs (61.9%), whereas seizures were more prevalent in those with DNETs (70%). The most frequent locations for the MVNTs were the frontal and parietal lobes (66.6%), while DNETs were most commonly located in the temporal lobe (60%). All the MVNTs were hyperintense in both fluid-attenuated inversion recovery (FLAIR) and T2-weighted imaging (T2WI). All the DNETs were hyperintense on T2WI. However, on FLAIR, seven (35%) of the DNET lesions were hyperintense, while the remaining 13 lesions showed mixed signal intensity forming a bubbly appearance. Moreover, 20 of 21 (95.23%) MVNTs were hyperintense on diffusion-weighted imaging (DWI) (b800), with no apparent diffusion coefficient hypointensity in the lesions. None of the DNETs showed hyperintensity on DWI.

**Conclusion:**

MRI findings, particularly those observed on FLAIR and DWI, may be helpful for distinguishing between MVNTs and DNETs, especially in cases where the differential diagnosis is challenging.

## 1. Introduction

Multinodular and vacuolating neuronal tumor (MVNT) is a rare, benign neuronal lesion with typical radiological and histological appearance [1,2]. This lesion was first defined approximately a decade ago and was included as a unique architectural pattern in the 2016 WHO Classification of Tumors of the Central Nervous System [3]. In the updated (2021) Fifth Edition of the WHO classification, it was recognized as a separate entity in the Glioneuronal and Neuronal Tumors group [4].

MVNTs are usually discovered as an incidental finding on magnetic resonance imaging (MRI), but in some cases, they may present with seizures or seizure equivalents [5]. Histologically they comprise tumor cells with remarkable stromal vacuolization arranged in nodules, mostly located in the temporal and parietal lobes with a predilection for subcortical-juxtacortical areas [6]. They are generally considered as ‘leave-me-alone’ lesions, showing no interval changes on the follow-up MRIs for years. Therefore, accurate and reliable diagnosis is important for avoiding misdiagnosis and unnecessary intervention. The primary differential diagnosis for MVNTs is dysembryoplastic neuroepithelial tumors (DNETs), another cortically-based benign lesion of glioneuronal-neuronal origin. DNETs are also seizure-related lesions and may have some imaging features that overlap with MVNTs [2,7].

Since MVNTs are rare and most cases have been described recently, there are limited data on their clinical and radiological features in the published literature. Moreover, there are no studies comparing the MRI features of MVNT and DNET lesions [2,7,8]. Hence, the aim of this study was to make a comprehensive characterization of the clinical and MRI features of MVNTs, and analyze the radiological differences from DNETs, which stand out as the main differential diagnosis.

## 2. Materials and methods

### 2.1. Ethics approval

This retrospective study was approved by the institutional review board and carried out according to the requirements of the Declaration of Helsinki. Informed consent was obtained from each participant included in the study group.

### 2.2. Patient selection

Data of patients with a suspected MRI-based diagnosis of MVNT between January 2018 and December 2022 were collected from the hospital database. In addition, patients diagnosed with DNETs on histopathological examination and who had MRIs in the same time period were included in the study. The DNET lesions were type 1a (cystic-like) and type 1b (polycystic-like) according to the classification introduced by Chassoux et al [9]. Patients with prior head trauma, who had undergone cranial surgery, had a history of chemotherapy/cranial radiotherapy, a intracranial accompanying mass lesion, a diagnosis of radiological subtype of two or three for DNET lesions, no follow-up MRI examinations or less than one year MRI follow-up for MVNT lesions that did not have histopathological confirmation were excluded from the study.

Consequently, 41 patients who met the inclusion criteria were included in the study and categorized into two groups: MVNT (21 patients) and DNET (20 patients). The diagnosis was histopathologically confirmed in three patients in the MVNT group. Demographic data and clinical findings were recorded for each patient.

### 2.3. MRI technique

MRIs were performed on either a 1.5 Tesla (Avanto; Siemens Healthcare, Erlangen, Germany) or a 3 Tesla scanner (Ingenia; Philips Healthcare, Best, Netherlands). The standard imaging protocol included sagittal turbo spin echo (TSE) T2, coronal TSE T2 (repetition time (TR): 3,000 ms, echo time (TE): 80 ms, field-of-view (FOV): 185 mm × 230 mm, matrix size: 400 × 320, slice thickness: 5 mm), axial fluid-attenuated inversion recovery (FLAIR) (TR: 11,000 ms, TE: 125 ms, inversion time: 2.5 s, FOV: 185 mm × 230 mm, matrix size: 370 × 260, slice thickness: 5 mm), axial T1-weighted imaging (T1WI) (TE: 17 ms, TR: 600 ms, matrix: 188 × 320, number of excitations: 1, FOV: 230 mm, flip angle: 90°), and diffusion-weighted imaging (DWI) sequences. In addition, gadolinium-enhanced T1WI sequences were performed in all the patients.

### 2.4. Radiological evaluation

MRI images of the MVNT and DNET lesions, including those at follow-up, were reviewed by two neuroradiologists (with 14 and 11 years of experience in neuroimaging) in a consensus analysis. The following major MRI features were evaluated for each lesion: location (frontal, parietal, temporal, or occipital lobes), size (overall size defined by the longest diameter of the lesion on FLAIR), signal intensity characteristics (compared with the normal appearing white matter), margins (defined as sharp or blurred), presence of enhancement on postcontrast T1WI, and presence of mass effect.

DWI characteristics of the lesions were first assessed as the presence or absence of a subjective diffusion restriction. Afterward, signal intensity characteristics of the lesions on DWI were evaluated and recorded as hyperintense, hypointense, or isointense. A mean apparent diffusion coefficient (ADC) value was calculated for each tumor using the following technique: regions of interest (ROIs) were placed manually. For optimal ADC quantification, FLAIR, DWI, and ADC maps were placed side by side, and three different ROIs were positioned within the tumor borders in different sections. Consequently, the mean ADC value was calculated by averaging the ADC values obtained from these three different regions of the lesion.

The following descriptive MRI features were also assessed: the nodularization pattern of the MVNT lesions (categorized as multiple small (nodules ≤5 mm in size), multiple large (nodules >5 mm in size), and mixed (both small and large nodules) patterns) [2], presence of cortical involvement for the MVNT lesions, presence of FLAIR hyperintense rim sign (a specific appearance with a thin rim of well-defined FLAIR hyperintensity surrounding the DNET, separating it from the surrounding normal brain) for the DNET lesions [10], and presence of a triangular pattern (a specific appearance defined as the tumor width being maximal at the cortex and decreasing toward the ventricles, best seen on coronal images) for the DNET lesions [11]. Extension of the lesions to the ventricle margin was also noted if present.

On the follow-up MRIs of the MVNT patients, the presence of a change in size was analyzed and it was accepted as a size change when a ≥5% change of the initial size was calculated [12]. Accompanying lesions such as multiple sclerosis plaques, etc., were noted as additional MRI findings when present.

### 2.5. Statistical analysis

All statistical analyses were performed using IBM SPSS Statistics for Windows 23.0 (IBM SPSS Statistics, Armonk, NY, USA). After the research data were digitized, frequency and percentage values were calculated for the categorical variables, and mean and standard deviation values were calculated for the continuous variables. Analyses were performed using the Student’s t test for the mean ADC measurements. Inter-reader agreement in the quantitative analysis was calculated utilizing intraclass correlation coefficients (ICCs) from a one-way random effects model analysis of variance, with the subject as the random effect. A 95% confidence interval (CI) was constructed for each ICC. An ICC >0.80 indicated excellent agreement between readers. Statistical significance was accepted as p < 0.05.

## 3. Results

The MVNT group included 14 females and seven males. Of the 20 patients in the DNET group, eight were female and 12 were male. The mean age was 37.9 ± 17.8 (range: 9–67) years in the MVNT group and 25.3 ± 14.2 (range: 1–59) years in the DNET group. Headache was the most common symptom in the MVNT group (61.9%), while seizures were more prevalent in the DNET group (70%). The clinical and demographic data of the MVNT and DNET patients are shown in Table 1.

**Figure 1:**
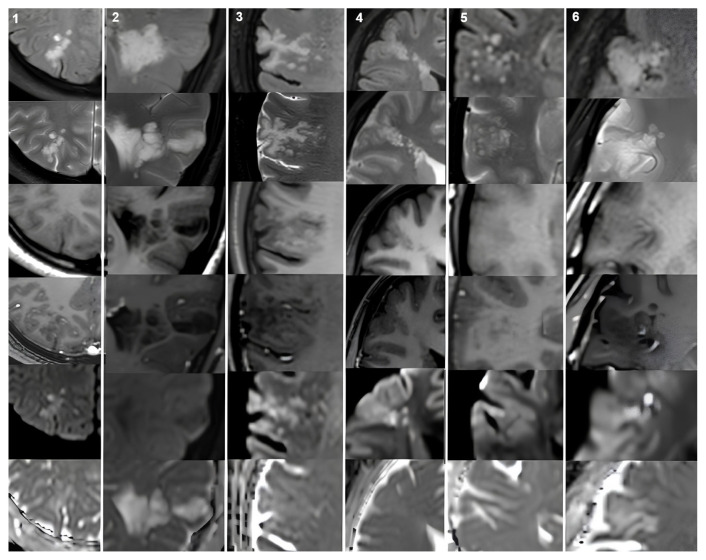
Representative MVNT cases. Each column contains MRI images of different cases. All the lesions are hyperintense on FLAIR and T2WI (first and second row). On precontrast T1WI, all the lesions are isointense compared to adjacent parenchyma except case 2. The MVNT lesion in case 2 has a hypointense signal on T1WI (third row). None of the lesions show contrast enhancement on postcontrast T1WI (fourth row). All the lesions except case 2 are hyperintense on b1000 DWI. The lesion in case 2 has an isointense signal compared to the adjacent parenchyma on DWI (fifth row). No hypointensity was observed on the ADC maps in any of the lesions (sixth row). In terms of the nodularization pattern, cases 1 and 4 had multiple small patterns, case 2 had multiple large patterns, and the remaining cases had a mixed pattern.

**Figure 2:**
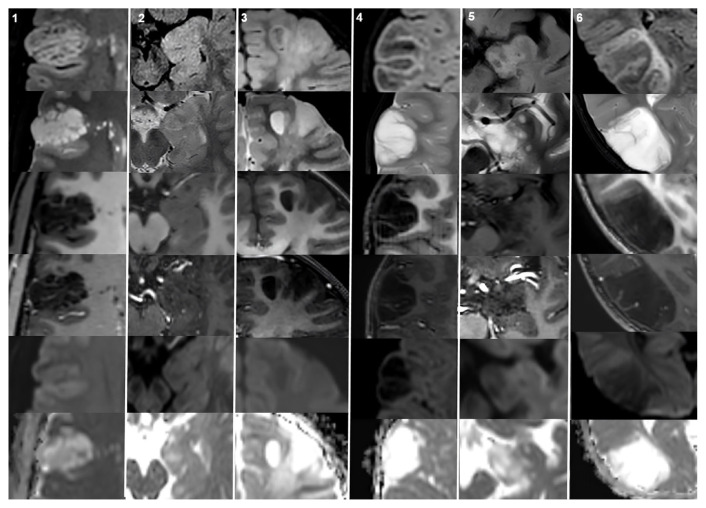
Representative DNET cases. Each column contains MRI images of different cases. All the lesions except case 2 have mixed signal intensity on FLAIR represented as high signal intensity with suppression of some internal areas. The lesion in case 2 has a hyperintense signal on FLAIR. Additionally, the FLAIR hyperintense rim sign was observed in cases 1, 4, and 6 (first row). All the lesions are hypointense on T1WI and hyperintense on T2WI (third and second row, respectively). None of the lesions in these six cases showed contrast enhancement on postcontrast T1WI (fourth row). On DWI, the lesions in cases 4 and 6 are hypointense, while the lesions in the remaining cases are isointense compared to the adjacent brain parenchyma (fifth row). No hypointensity was observed on the ADC maps in any of the lesions (sixth row).

The most frequent locations for the MVNTs were the frontal and parietal lobes (14/21, 66.6% of patients), whereas the DNETs were most commonly located in the temporal lobe (12/20, 60% of patients). In terms of the lesion size, the mean maximum diameter was 23.1 ± 8.7 (range: 9–39) mm in the MVNT group and 23.1 ± 8.6 (range: 10–40) mm in the DNET group. Moreover, 19 of the 20 DNETs (95%) were Type1b, while the remaining lesions were Type 1a. All the MVNT lesions were hyperintense on both the FLAIR and T2WI. All the DNET lesions were hyperintense on T2WI. However, on FLAIR, seven (35%) of the DNET lesions were hyperintense, whereas the remaining 13 lesions showed mixed signal intensity, represented as high signal intensity with the suppression of some internal areas, forming a bubbly appearance. In eight of the 20 (40%) DNET lesions, a FLAIR hyperintense rim sign was observed, while no such rim was seen in the MVNT lesions. On the gadolinium-enhanced T1WI sequences, none of the MVNT lesions showed contrast enhancement and only one (5%) DNET lesion showed minimal mural contrast enhancement (Figures 1 and 2). No mass effect was observed in either the MVNT or DNET lesions. The cerebral cortex was involved in nine (43%) of the 21 MVNT lesions, while the remaining 12 lesions were located in the subcortical/juxtacortical region.

None of the MVNT and DNET lesions showed diffusion restriction on DWI, with a mean ADC of 0.99 ± 0.18 × 10^−3^ and 1.68 ± 0.34 × 10^−3^ mm^2^/s, respectively. Interobserver agreement was found to be excellent for the mean ADC values, at 0.918 (0.831–0.951). Moreover, 20 of the 21 (95.23%) MVNT lesions were hyperintense on DWI (b800) compared to the adjacent brain and no hypointensity was observed on the ADC maps, indicating diffusion restriction (Figure 1, fifth and sixth rows). On the other hand, none of the lesions showed DWI hyperintensity in the DNET group (Figure 2, fifth row). There was a statistically significant difference between the two groups (p = 0.021). Detailed MRI characteristics are presented in Table 2. The features of the MVNTs and DNETs on different MRI sequences are shown in Figures 1 and 2, respectively.

On histopathological evaluation, two patients with DNETs had cortical dysplasia accompanying the lesion. Of the three MVNT patients whose diagnosis was histopathologically confirmed, one patient had cortical dysplasia accompanying the MVNT and the presenting symptom was seizures. In two MVNT patients, previously diagnosed as multiple sclerosis, accompanying demyelinated plaques were observed.

Eighteen MVNT patients (85.7%) had follow-up MRIs and the median follow-up period was 33 (range: 12–56) months. The remaining three MVNT patients underwent surgery after the initial MRI, conﬁrming the diagnosis.

## 4. Discussion

The clinical and MRI features of MVNT cases were evaluated in detail and compared with those of DNETs, which was the main differential diagnosis. Given the markedly different treatment regimens and prognoses, it is crucial to make a distinction between MVNTs and DNETs. MVNTs are considered benign lesions without any potential for malignancy and evolution unless they cause seizures. Thus, these lesions can be sufficiently managed on follow-up MRIs [2]. On the other hand, surgical treatment is essential following the diagnosis in DNETs [13].

The primary imaging modality for the diagnosis of MVNTs is MRI, and these lesions have a distinctive characteristic appearance. MVNTs have lesions with a bubbly appearance located in the deep cortical ribbon and superficial subcortical white matter. They are observed as hyperintense on both T2WI and FLAIR sequences and do not enhance. They also do not show edema or mass effect [1,2,6]. Similarly, DNETs are lesions that are hyperintense on T2WI and FLAIR, do not enhance in most cases, and do not cause mass effect [9,11,14]. Subtypes 1a and 1b, which were also included in the current study, are defined as cystic/polycystic-like and may have a soap bubble appearance, mimicking MVNTs [9]. Thus, the overlapping imaging findings can make the differentiation of MVNT and specific DNET subtypes challenging. Therefore, the differential diagnosis of MVNTs and type 1 DNET lesions were focused on in the present study.

One of the features that helps to distinguish DNETs from MVNTs is cortical involvement [7,15]. However, cortical involvement has been reported in up to 57% of MVNTs [7]. In the current study, cortical involvement of MVNTs was found at a rate of 42.8%. Moreover, although DNETs are mainly cortical-based lesions, they also involve the adjacent white matter at varying degrees [16]. Consistently, extension from the cortex to the subcortical white matter was observed in all of the DNET cases in the present study. This revealed that cortex and white matter involvement may not be reliable in differentiating MVNTs from DNETs in cases with overlapping MRI findings.

The FLAIR hyperintense rim sign is a well-known specific appearance in DNETs [5,7,10,17]. It is also speculated that the presence of this ring on postoperative imaging indicates a residual or recurrent tumor [10]. In a study by Isler et al. [17], this was found in four (19%) of the 21 DNET patients. However, Parmar et al. [10] reported that nine of the 11 DNET patients and two of 21 of those in the non-DNET control group displayed this sign, resulting in a high sensitivity and specificity for DNETs, as 82% and 90% respectively. Herein, it was detected in 8 (40%) of the 20 DNET patients, while no such rim was demonstrated in the MVNT patients, resulting in a sensitivity of 40% and specificity of 100%.

Another feature that may facilitate the differentiation of MVNTs from DNETs on FLAIR sequences is the signal suppression in some parts of the lesion detected in DNETs. Previous reports have suggested that this signal suppression observed on FLAIR sequences is a remarkable imaging feature in DNETs. In addition, signal suppression in some parts of the lesion allows better demonstration of the ‘bubbly’ appearance [11,14]. In the current study, signal suppression in some focal areas of the lesion and mixed signal intensity were observed in 13 of the 20 DNET lesions on the FLAIR sequences. On the other hand, no signal suppression was observed on the FLAIR sequences in the MVNT group.

Thus far, studies have determined that MVNTs do not show diffusion restriction, reflecting the hypocellular nature of the tumor [5,7,12]. Several data regarding the measurement of ADC in MVNTs have been reported in the literature, and Lecler et al. [12] reported a slightly increased median ADC value of 1.13 × 10^−3^ mm^2^/s, compatible with the previous studies [1,2,7]. DNETs, similar to MVNTs, do not show diffusion restriction, but were reported to have high relative ADC values [18]. In a study by Daghistani et al. [15], the relative mean ADC values of two DNET groups were calculated as 2.22 ± 0.59 × 10^−3^ and 2.21 ± 0.74 × 10^−3^ mm^2^/s. The current results were the similar to those in the literature, as none of the MVNT and DNET lesions showed diffusion restriction. The median relative ADC values for the MVNT and DNET groups herein were 0.99 ± 0.18 × 10^−3^ and 1.68 ± 0.34 × 10^−3^ mm^2^/s respectively. Furthermore, the DWI intensity of each lesion in the MVNT and DNET groups were also evaluated. Noticeably, in 20 (95%) of the 21 MVNT patients, lesions showed high DWI signal intensity compared with the normal appearance of the white matter without associated hypointensity on the ADC maps. However, none of the DNET lesions showed hyperintensity on DWI. In studies by Lecler et al. [12] and Nunes et al. [2], mild high signal intensity on DWI was mentioned for some particular cases.

Clinical presentation and lesion location may aid in the differentiation between DNETs and MVNTs. Most of the DNET cases in the published literature presented with drug-resistant epilepsy. On the other hand, MVNTs are often detected incidentally on MRI studies [11,19]. In symptomatic MVNT cases, the most common symptoms are headache and seizures [8,20]. In the present study, the most dominant clinical presentation in the DNET cases was seizure (70%). Similar to the literature, headache (13/21) and seizure (3/21) were the most common symptoms in the MNVT cases.

The most common location of DNETs is the temporal lobe (62%), followed by the frontal lobe (31%). On the other hand, in MVNTs, the lesion location is not consistent in different studies [1,12]. Lecler et al. [12] reported that MVNTs were mostly located in the frontal and parietal lobe. In a study conducted by Huse et al. [1], the most common location was reported as the temporal lobe. In addition, in some cases, more than one lobe may be affected [20]. In the current MVNT group, there were similar rates of involvement in the frontal, parietal, and temporal lobes. Moreover, in three cases, more than one lobe was affected.

This study had several limitations. First, it was conducted on a limited number of patients and had a retrospective design. Second, the diagnosis was histopathologically confirmed in only three of the patients in the MVNT group in order to provide a persistent seizure-free state. However, no interval changes were observed in the follow-up MRIs of the MVNTs that were not confirmed histopathologically, for at least 12 months.

## 5. Conclusion

MVNT is a rare and stable tumor, which is generally considered as a ‘leave-me-alone’ lesion showing no interval changes on follow-up MRIs. Surgical removal of MVNT is unnecessary unless it is symptomatic. Detailed characterization of its diagnostic and discriminative MRI features, which enables the differentiation from DNET, has substantial importance for the accurate diagnosis and the avoidance of unnecessary surgical interventions.

## Figures and Tables

**Table 1:** Demographic and clinical data of the MVNT and DNET patients.

	MVNT (n = 21)	DNET (n = 20)
Age (mean, year)	37.90 ± 17.83	25.35 ± 14.28
Sex (female/male)	14:7	8:12
Clinical presentation		
Headache	13 (61.9%)	5 (25%)
Seizures	3 (14.2%)	14 (70%)
Memory impairment	1 (4.76%)	0 (0%)
Vertigo	1 (4.76%)	0 (0%)
Multiple sclerosis follow-up	2 (9.52%)	0 (0%)
Extremity weakness	1 (4.76%)	1 (5%)

MVNT: multinodular and vacuolating neuronal tumor, DNET: dysembryoplastic neuroepithelial tumor.

**Table 2:** MRI features of the MVNT and DNET groups.

	MVNT (n = 21)	DNET (n = 20)

Location		

Frontal lobe	7 (33.33 %)	5 (25%)

Parietal lobe	6 (28.57%)	3 (15%)

Temporal lobe	5 (23.83%)	12 (60%)

Occipital lobe	0 (0%)	0 (0%)

Frontal + parietal lobe	1 (4.76%)	0 (0%)

Temporal + parietal lobe	1 (4.76%)	0 (0%)

Temporal + occipital lobe	1 (4.76%)	0 (0%)

Size (mm)	23.19 ± 8.77	28.9 ± 8.06

Signal intensity		

T1WI		
Hypointense	2 (9.52%)	20 (100%)

Isointense	19 (86.38%)	0 (0%)

Hyperintense	0 (0%)	0 (0%)

T2WI		
Hypointense	0 (0%)	0 (0%)

Isointense	0 (0%)	0 (0%)

Hyperintense	21 (100%)	20 (100%)

FLAIR		
Hypointense	0 (0%)	0 (0%)

Hyperintense	21 (100%)	7 (35%)

Mixed	0 (0%)	13 (65%)

Postcontrast T1WI		

Enhancement		
Mass effect	0 (0%)	1 (5%)
Yes	0 (0%)	0 (0%)
No	21 (100%)	21 (100%)

DWI		

Hyperintense	20 (95.24%)	0 (0%)

Isointense	1 (4.76%)	12 (60%)

Hypointense	0 (0%)	8 (40%)

ADC (× 10^−3^ mm^2^/s) (mean ± SD)	0.99 ± 0.18	1.68 ± 0.34

Type of margins		

Sharp	21 (100%)	20 (100%)

Blurred	0 (0%)	0 (0%)

Nodularization pattern		

Multiple small	9 (42.85%)	-

Multiple large	2 (9.52%)	-

Mixed	10 (47.63%)	-

FLAIR hyperintense rim sign		

Yes	0 (0%)	8 (40%)

No	0 (0%)	12 (60%)

Triangular pattern		

Yes	0 (0%)	4 (20%)

No	21 (100%)	16 (80%)

Extension to the ventricle margin		

Yes	2 (9.53%)	2 (10%)

No	19 (90.47%)	18 (90%)

ADC: Apparent diffusion coefficient.
